# Delayed Development of Head Control and Rolling in Infants With Tracheostomies

**DOI:** 10.3389/fped.2020.571573

**Published:** 2020-10-30

**Authors:** Hyun Iee Shin, Hyung-Ik Shin

**Affiliations:** ^1^Department of Rehabilitation Medicine, Chung-Ang University College of Medicine, Seoul, South Korea; ^2^Department of Rehabilitation Medicine, Seoul National University Hospital, Seoul National University College of Medicine, Seoul, South Korea

**Keywords:** tracheostomy, development, head control, rolling, gross motor function measure

## Abstract

**Objective:** Advances in neonatal care lead to an increased survival rate of critically ill babies. Infantile tracheostomies are not uncommon. However, only a few studies have addressed the effect of infant tracheostomy on early motor function. By comparing the scores of the Gross Motor Function Measure-88 (GMFM) on head control and rolling of infants with and without tracheostomies, the authors aimed to evaluate the effect of infant tracheostomy on early motor development.

**Methods:** Medical records and the GMFM of subjects were retrospectively reviewed. Thirty-three infants with tracheostomies and 132 infants without tracheostomies were matched by gestational age, birth weight, and corrected age when the GMFM was performed using propensity score matching. GMFM scores in head control and rolling in different positions were compared by using generalized estimating equation (GEE).

**Results:** Infants with tracheostomy showed lower values for head control in the supine position and in the pull to sit maneuver in multivariate GEE (*p* = 0.008, 0.004, respectively). However, the results of head control in a prone position and head lift while the examiner held the thorax showed no difference between the groups. Rolling from prone to supine was delayed in the infants with tracheostomy (*p* = 0.002), while rolling from supine to prone was not delayed compared to the non-tracheostomized group. More than half (54%) of the tracheostomy group scored better in rolling from a prone to supine position than in head control in supine position, which was a higher ratio compared to the non-tracheostomy group (*p* = 0.00).

**Conclusions:** Tracheostomy seems to influence early motor development in infants. In particular, head control skills related to neck flexor muscle activation and rolling from prone to supine were delayed. Interventions may be required to facilitate these activities.

## Introduction

Due to advances in preterm neonatal care, the survival rate of very premature and low birth weight infants has increased (1–4). In the same context, care of critically ill babies with congenital anomalies has also greatly improved (5). This in turn has led to an increased rate of tracheostomies in infants (1, 4). Tracheostomies in infants are known to reduce the need for a long period of intubation, thereby reducing any risks of tracheal stenosis. It is also known that tracheostomy enables improved nutrition and growth, as it gives more comfort to the oropharyngeal system, allowing oral feeding (6). However, it is also thought to be associated with adverse neurodevelopmental outcomes (7, 8).

There were prior studies that reported the delay in development of children with tracheostomies. Most studies have noted impairments in speech and language, intelligence, or physical growth (6, 8–13). Sisk et al. (6) showed that tracheostomized infants had higher incidences of disorders of speech, language, and swallowing. Singer et al. documented that children who had had tracheotomies showed lower intellectual functioning, language skills, growth measurements such as weight, height, and head circumference, and even behavioral problems (9). However, motor function was not studied in depth in this patient group. Demauro et al. (10) reported that infants who underwent tracheostomies showed adverse developmental outcomes, which was evaluated using the Bayley–III. However, they only presented the total scores and did not analyze the results separately from the gross motor function domain of the Bayley-III.

Head control and rolling are the earliest motor developmental skills that an infant acquires (14). Head control is a basic skill that is thought to be a cornerstone for more advanced motor development. After head control is achieved, infants are able to transit from one posture to another, a skill known as axial rotation, or rolling (15). On the other hand, the tracheostomy tube itself, located on the ventral side of neck, can hamper neck flexion motion mechanically. Tracheostomy is frequently associated with devices such as ventilators, which can further interrupt the neck motion of patients in other directions, including neck extension and rotation; this in turn may also affect the development of rolling.

The Gross Motor Function Measure (GMFM) is a common functional gross motor outcome measure (16). In GMFM, head control is evaluated in four different positions: head lifting in a supine position and a prone position, head lifting while pulling a patient to a sitting position, and head lifting in a sitting position with the thorax supported by the examiner. Rolling is defined as moving from a supine to a prone, and from a prone to a supine position.

The authors aimed to investigate how tracheostomy affects early motor development in infants. Therefore, we compared the GMFM scores related to head control and rolling in infants with and without tracheostomies.

## Materials and Methods

### Subjects

We retrospectively reviewed medical records of tracheostomized infants who were referred to the Pediatric Rehabilitation division between March 2013 and February 2018. Sixty-two patients who had undertaken a GMFM assessment within at least one month from the date of the tracheostomy were included in the study (9). As we targeted the early motor development of the infants, GMFM taken at > 2 years of corrected age were also excluded from the study. Infants with syndromic conditions, brain anomaly or lesion, and neuromuscular disorder that could influence the early motor development were excluded.

After the tracheostomy group had been identified, the control group was defined as infants without tracheostomies but with GMFM results. The research was conducted in accordance with the principles of the Declaration of Helsinki and was approved by Institutional Review Board (IRB No. 1804-169-942).

### Propensity Score (PS) Matching

To control for the nonrandom assignment of patients, we undertook a PS-matched analysis to construct a weighted cohort of patients who differed by existence of tracheostomy, but were similar with respect to other measured characteristics (17). The PSs were estimated using multiple logistic-regression analysis. Predictor variables had been selected on the basis of their potential to confound the relationship between motor development and infant tracheostomy; these variables include gestational age (GA), birth weight (BW), and corrected age when GMFM was performed. Matching was performed in a 1:4 ratio using the greedy matching algorithm. To ensure close matches, a caliper width of 0.2 of standard deviation of the logit of PS was used. This minimizes the mean square error of the resultant estimated treatment effect and eliminates at least 98% of the bias in the crude estimator (18).

### GMFM Assessment

Korean version Gross motor function measure-88 (K-GMFM-88) was used (19). The assessment was performed according to the “Administration and Scoring Guidelines for the GMFM-88 and the GMFM-66” in the GMFM User's manual (20). We administered the assessments in a pediatric physical therapy room that was familiar to the subjects. All children were assessed barefoot, without assistive devices. The procedure was performed by two physical therapists. Both of them had more than five years of experience in the evaluation and the treatment of children with developmental delay.

Among the five dimensions of GMFM, lying and rolling (dimension A), and sitting (dimension B) were reviewed for the evaluation of early gross motor function in the infants.

To evaluate head control in different positions, four items were selected; in dimension A, item 3 (supine, lifts head 45°) and item 10 (prone, lifts head upright) were used, and in dimension B, item 18 (supine, hands gasped by examiner: pulls self to sitting with head control) and item 21 (sits on mat, supported at thorax by therapist who lifts head upright, maintains 3 s) were evaluated.

For the evaluation of rolling function, items 8 and 9 (supine: rolls to prone over right and left side, respectively), and items 14 and 15 (prone: rolls to supine over right and left side, respectively) in dimension A were selected. Since each functional measure in rolling is shown on either side, right, and left, a higher score was used in the evaluation to approximate the best possible ability of each subject.

To compare the overall developmental status of the two groups, total GMFM score was also investigated. The total score for the GMFM-88 was based on the percentages for the five domains.

The GMFM scoring key ranges from 0-3. 0 is defined as “does not initiate,” 1 as “initiates,” 2 as “partially completes,” 3 as “completes,” and NT as “not tested.” However, each item has its own specific descriptors (21) according to its specific function (Supplementary Table 1).

### Review of Medical Records

The authors collected each participant's inpatient and outpatient medical records. Functional ability classified by the Gross Motor Function Classification System (GMFCS) (22) was collected. It was assessed by the two physical therapists. Those who had a central nervous system (CNS) diagnosis such as a minor brain anomaly or brain injury and periventricular echogenicity (PVE) grade higher than 3 were classified in the CNS abnormality category. The presence or absence of prematurity (gestational age ≤ 36 weeks), and complications of prematurity, such as bronchopulmonary dysplasia (BPD), necrotizing enterocolitis (NEC), and retinopathy of prematurity (ROP) were reviewed. Reporting of findings and diagnosis was originated from the information documented in the charts of the department of pediatrics. Definition of BPD was adapted from NIH consensus (23). Histories of surgeries, diagnosis of hypothyroidism, sepsis, and heart anomaly were also reviewed. One and 5 min Apgar scores were investigated. Feeding history was analyzed, those who had histories of parenteral feedings were classified into partial oral feeding group and non-oral feeding groups.

### Statistical Analysis

Distributions of variables in the tracheostomized group and in the non-tracheostomized group were compared using Mcnemar's test for categorical variables and mixed model analysis for continuous variables.

GMFM scores between the two groups were compared using a generalized estimating equation (GEE). As multiple control subjects were matched to a single study group subject, correlation among data was considered. To adjust for factors that may affect the development of participants, multivariate GEE was used. Adjustment factors were selected as factors that are believed to affect a child's development when GMFM was performed; these include the existence of tracheostomy, the existence of BPD, a CNS abnormality, and sepsis. All statistical analyses were conducted with the SPSS version 25.0 software (IBM SPSS Statistics, IBM Corporation, Armonk, NY).

## Results

### Characteristics of Participants

**Figure 1:**
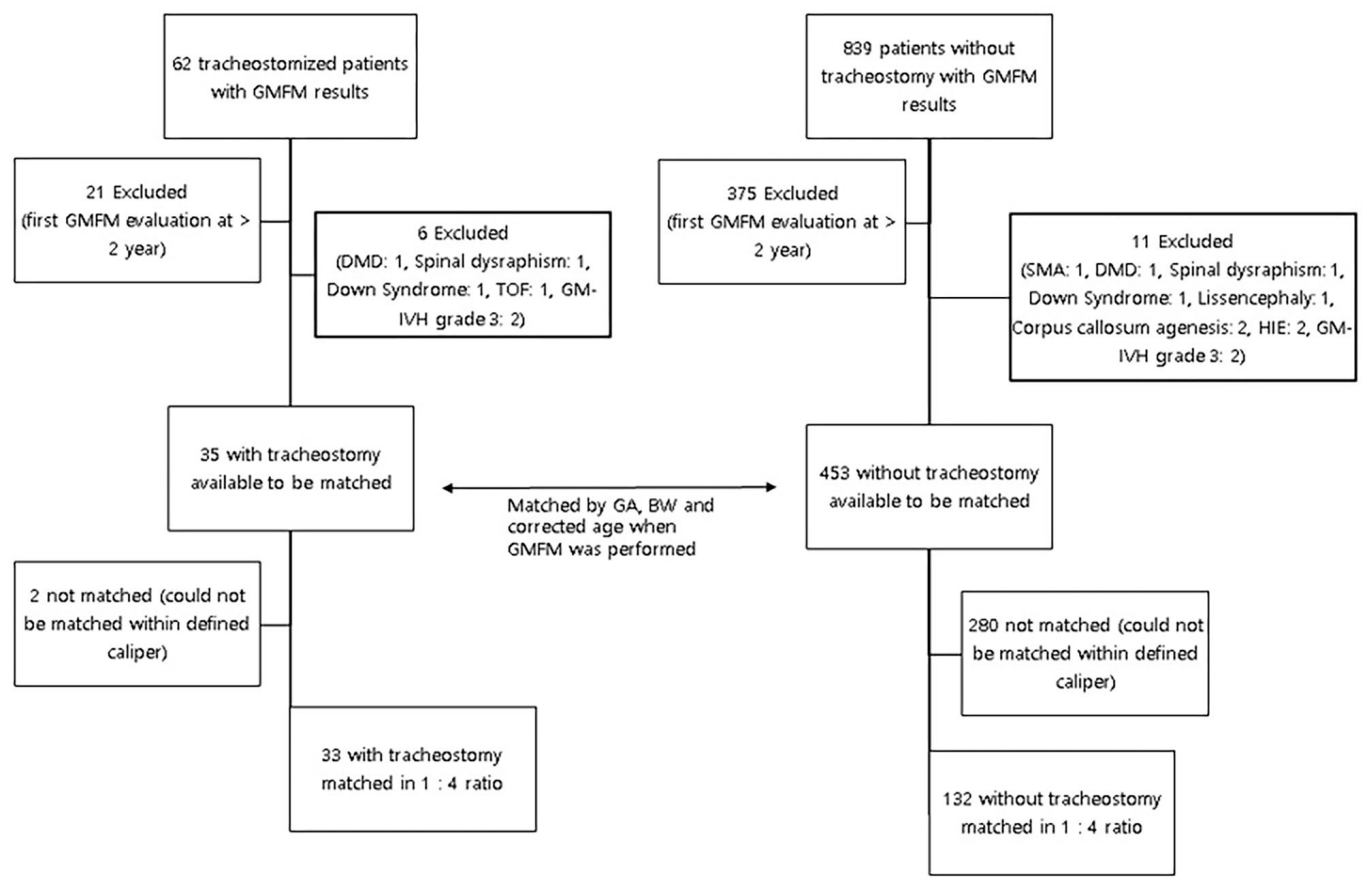
Process for matching in infants with and without tracheostomies. GMFM, Gross motor function measure; DMD, Duchenne muscular dystrophy; TOF, Tetralogy of Fallot; GM-IVH, Germinal matrix-inraventricular hemorrhage; HIE, Hypoxic ischemic encephalopathy; GA, gestational age; BW, Birthweight.

Thirty-three participants with tracheostomy and 132 participants without tracheostomy were included. In each group with infantile GMFM results, there were 6 subjects in the tracheostomy group and 11 subjects in the control group with the diagnosis that was thought to affect the early infantile motor function, and therefore excluded from the analysis (Figure 1). There was no difference between the study and control group in basal characteristics except the existence of NEC (*p* = 0.025, Table 1). Indications of tracheostomy are shown in the tracheostomy group (Supplementary Table 2). Many of the subjects were tracheostomized because of the prolonged intubation due to bronchopulmonary dysplasia.

**Table 1:** Demographic and developmental characteristics of subjects.

**Characteristics**		**Total (*N* = 165)**	**Tracheostomy (*N* = 33)**	**No tracheostomy (*N* = 132)**	***p*-value**
Sex	Male	95	20	75	0.815
	Female	70	13	57	
GA	GA (weeks)	31.7	32.6 ± 5.6	31.7 ± 6.1	0.359
	Preterm	112	20	92	1.000
	Full term	53	13	40	
BW (g)		1,749.5 ± 1,024.2	1,831.6 ± 1,020.6	1,729.0 ± 1,027.9	0.608
Corrected age (month) at GMFM		11.5 ± 9.5	12.6 ± 6.3	10.3 ± 12.7	0.488
GMFM total score		45.4 ± 24.5	46.0 ± 30.8	45.2 ± 22.8	0.138
GMFCS		4.2 ± 0.7	4.2 ± 1.0	4.2 ± 0.6	0.869
BPD		73	18	55	0.053
Minor brain abnormality^†^		73	19	54	0.110
NEC		17	0	17	0.025^*^
Sepsis		20	1	19	0.375
GI surgeries^†^		70	11	59	0.237
Heart anomaly^†^		80	20	60	0.172
Feeding	Full oral feeding	104	25	79	0.090
	Partial oral feeding	39	6	33	0.519
	Non-oral feeding	30	2	28	0.795
Apgar score	1 min	4.27 ± 2.03	3.70 ± 2.11	4.83 ± 1.94	0.536
	5 min	6.53 ± 1.72	6.40 ± 2.41	6.66 ± 1.03	0.669
ROP		47	6	41	0.196
Hypothyroidism		28	6	22	0.836

*Values are presented in number of subjects, and as mean ± standard deviation*.

*Categorical variables were compared using Mcnemar's test. Continuous variables were compared using mixed model analysis*.

† *Specific diagnosis and number of the subjects in each group are listed in the Supplementary Table 3*.

*GA, Gestational age; BW, Birth weight; GMFM, Gross Motor Function Measure; BPD, Bronchopulmonary dysplasia; CNS, Central nervous system; NEC, Neonatal enterocolitis; GI, gastrointestinal; ROP, Retinopathy of Prematurity*.

### GMFM Score of Early Motor Development; Head Control, and Rolling

The total GMFM score and GMFCS level of the participants were not different between the groups (Table 1).

In the head control dimension of GMFM, the tracheostomy group showed a lower score in the head lift at a supine position (*p* = 0.002). The tracheostomy group also showed delayed development in head control in the pull to sit maneuver (*p* = 0.002). However, the head lift in prone position, and head control in sitting position with thorax supported by examiner did not show any difference between groups. In the rolling dimension of GMFM, scores of rolling from prone to supine position were different between two groups (*p* = 0.002), while rolling from supine to prone position did not show a significant difference (Table 2). In multivariate GEE, when adjusted for other factors, the existence of tracheostomy was significant for the following GMFM items: head lift at supine position, head lift in pull to sit maneuver, and rolling from prone to supine (*p* = 0.008, 0.004, and 0.002, respectively; Table 3).

**Table 2:** Gross motor function measure value of items of head control and rolling between tracheostomy and no tracheostomy groups.

**Dimension**	**Item**	**Score**	**With tracheostomy (*N* = 33)**	**No tracheostomy (*N* = 132)**	***p*-value**
Head control	Supine, lifts head 45°	0	10 (30.3)	30 (22.7)	0.002^*^
		1	12 (36.4)	24 (18.2)	
		2	6 (18.2)	15 (11.4)	
		3	5 (15.2)	63 (47.7)	
	Prone, lifts head upright	0	3 (9.1)	23 (17.4)	0.242
		1	6 (18.2)	11 (8.3)	
		2	11 (33.3)	32 (24.2)	
		3	13 (39.4)	66 (50.0)	
	Pulls self to sitting with head control	0	21 (63.6)	73 (55.3)	0.002^*^
		1	3 (9.1)	12 (9.1)	
		2	1 (3.0)	12 (9.1)	
		3	8 (24.2)	35 (26.5)	
	Sit on mat, supported at thorax by therapist, lifts head upright, maintains 3 s	0	28 (84.8)	122 (92.4)	0.104
		1	3 (9.1)	5 (3.8)	
		2	1 (3.0)	2 (1.5)	
		3	1 (3.0)	3 (2.3)	
Rolling	Supine: rolls to prone	0	4 (12.1)	22 (16.7)	0.634
		1	6 (18.2)	14 (10.6)	
		2	12 (36.4)	32 (24.2)	
		3	11 (33.3)	64 (48.5)	
	Prone: rolls to supine	0	19 (57.6)	49 (29.7)	0.002^*^
		1	3 (9.1)	16 (9.7)	
		2	5 (15.2)	19 (11.5)	
		3	6 (18.2)	48 (29.1)	

*Values are in number of participants (%)*.

* *p-value < 0.05*.

**Table 3:** Multivariate general estimating equation according to each item.

**GMFM item**	**Characteristics**	**Estimate (95% CI)**	***p*-value**
Supine, lifts head 45°	Tracheostomy	1.47 (−0.27 to 1.73)	0.008^*^
Prone, lifts head upright	Tracheostomy	0.29 (-0.13 to 0.69)	0.177
Supine, pulls self to sitting with head control	Tracheostomy	0.71 (0.22 to 1.20)	0.004^*^
	Brain injury	0.41 (0.04 to 0.78)	0.029^*^
Sit on mat, supported at thorax by therapist lifts head upright, maintains 3 s	Tracheostomy	0.022 (−0.509 to 0.553)	0.935
	Brain injury	0.54 (0.17 to 0.92)	0.004^*^
	BPD	0.15 (0.01 to 0.29)	0.049^*^
Supine: rolls to prone	Tracheostomy	0.10(−0.27 to 0.47)	0.600
	Brain injury	0.54(0.23 to 0.85)	0.001^*^
Prone: rolls to supine	Tracheostomy	0.65(0.23 to −1.07)	0.002^*^
	Brain injury	0.66(0.31 to 1.01)	0.000^*^

*GMFM, Gross motor function measure; CI, Confidence interval; BPD, Bronchopulmonary dysplasia*.

* *p-value < 0.05*.

### Sequences of Head Control and Rolling Activities

**Figure 2:**
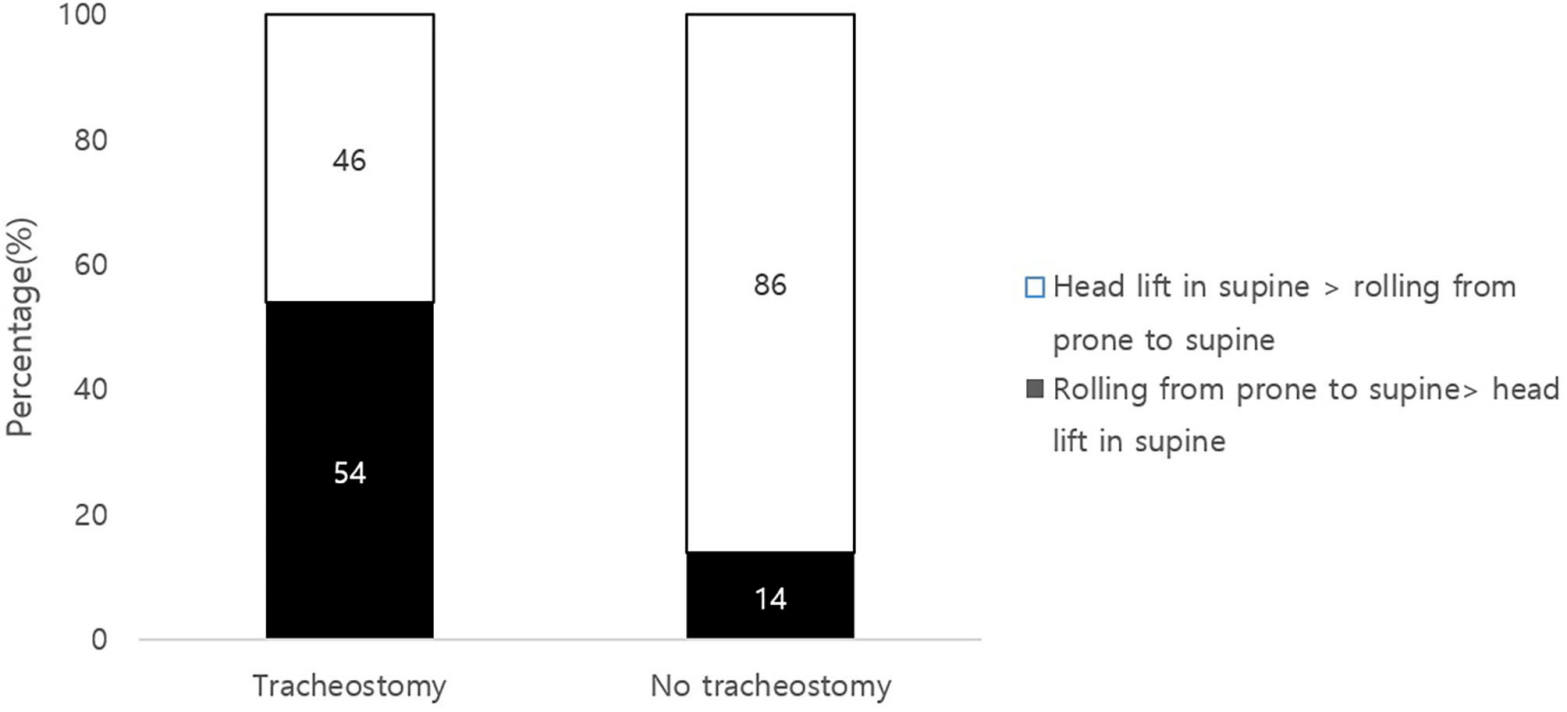
Comparisons of head control and rolling scores in the tracheostmy to non-tracheostomy group. Scores of GMFM item of head lift in supine position and score of rolling from prone to supine position were compared. Note that 54% of the tracheostomy group scored better in rolling than a head control (black). Head control precedes rolling in typical developmental milestones.

More than half (54%) of the tracheostomy group scored better in rolling from a prone to supine position than from a head control in supine position, which was a higher ratio compared to the non-tracheostomy group (*p* = 0.00; Figure 2). Other combinations of comparing the sequences in head control in different positions and rolling did not show a significant difference between the groups. The tracheostomized group scored better in supine to prone rolling than in prone to supine rolling maneuver; however, the non-tracheostomy group scored higher in prone to supine positions (*p* = 0.031, 0.042, respectively).

## Discussion

In this study, infant tracheostomy had a significant influence on head control in a supine position, in the pull to sit maneuver, and in the rolling from prone to supine position. It should be noted that rolling and head lifting in other positions were not significantly different between the two groups. If the difference were merely due to a developmental delay in the tracheostomized group owing to medical conditions or other abnormalities, head control, and rolling in other positions would also show a difference between the two groups. The gross motor function classification system (GMFCS) level and total value of the GMFM score did not show a difference between the two groups.

Head control in a supine position and in the pull to sit maneuver are mainly associated with neck flexor muscle activation (24, 25). Therefore, the authors assumed that tracheostomized infants are especially vulnerable to delay in neck flexor activation due to a mechanical disturbance of the tube. Other head control skills, such as head lifting in prone position or head lifting in sitting position by the examiner holding the thorax, involve neck extensor muscle activation and there were no differences between the two groups. The authors speculated that tracheostomy can have a mechanical disturbance on the patient's neck flexion, as the tube is placed on the anterior portion of neck. Additionally, Head control is known to be the first gross motor developmental milestone that a human being acquires. It precedes other motor skills, and is also known to be a prerequisite for other motor functions such as rolling, sitting, and reaching (26–28). However, as shown in Figure 2, it was noticeable that compared to the non-tracheostomized group, 54% of the tracheostomized infants did not follow the routine developmental sequence of achieving head control followed by rolling (26–28).

Our study also showed a difference between the two groups in rolling function. A possible explanation for this difference is that tracheostomized infants may not have spent enough time in prone positions. In the traditional motor milestone sequence, rolling from a prone to a supine position precedes rolling from a supine to a prone position (29–31). However, the tracheostomized group in this study scored higher in supine to prone rolling than in prone to supine rolling.

It is widely known that prone positioning has a positive impact on motor development in infants (21, 31–33). It is also reported that babies who regularly play in a prone position achieve motor functions more rapidly than those who do not (21, 34). While our study showed that most infants with tracheostomy showed delayed development in head control in a supine position and rolling from a prone to a supine position, we cannot ascertain whether developmental progression and further motor development are affected by this delay in specific motor skills. However, based on the results of this study, clinicians could propose a method to support early motor development of infants with tracheostomies.

Exercise programs should be targeted on strengthening the neck flexor muscle. Gently holding the baby's hands and wrists to pull them up slowly from a supine position can be a good ventral neck muscle exercise (35). Holding babies' attention with toys while they are in a supine position can be another good option (36). Clinicians should also check whether tracheostomized infants spend enough time in a prone position. If parents report that they are afraid that a prone position might interfere with the tracheostomy tube or connected ventilators, prone wedges or supports could be recommended.

There are some limitations in this study. Due to its retrospective nature, no follow-up GMFM or other functional outcomes were measured during the study. Additional research is recommended so that a functional outcome could be assumed; this would help determine whether this developmental aberration leads to eventual developmental delay, or whether it could be overcome during the developmental process. As a result, future prospective studies with follow-up GMFM results are necessary. Also, there are many other factors that could influence the development of a child. It is widely accepted that lower socioeconomic status (SES) including parental education level or marital status influences the well-being and development of children (33, 34). However, caregiver factors and socioeconomical status have not been investigated in this study.

Infants with tracheostomy showed delayed gross motor milestones in head control in a supine position and rolling from a prone to a supine position compared to non-tracheostomized infants. These findings could help clinicians establish targeted intervention plans for this patient group to enhance early motor development.

## Data Availability Statement

The raw data supporting the conclusions of this article will be made available by the authors, without undue reservation.

## Ethics Statement

The studies involving human participants were reviewed and approved by IRB No. 1804-169-942. Written informed consent for participation was not provided by the participants' legal guardians/next of kin because: This was a retrospective study.

## Author Contributions

HS conceived the study. HS and H-IS drafted the manuscript. H-IS critically revised the manuscript for important intellectual content. All authors have read and approved the manuscript and agreed to be accountable for all aspects of the work in ensuring that questions related to the accuracy or integrity of any part of the work are appropriately investigated and resolved.

## Conflict of Interest

The authors declare that the research was conducted in the absence of any commercial or financial relationships that could be construed as a potential conflict of interest.
