# Supplementary material for: Delayed Development of Head Control and Rolling in Infants With Tracheostomies
Source: Front Pediatr. 2020 Oct 30;8:571573. doi: 10.3389/fped.2020.571573 (PMC7661431; doi:10.3389/fped.2020.571573)
Supplement: Supplementary file 1 [file Table_1.DOCX]

Supplementary table 1. Gross motor function measure (GMFM) items according to functions

| Function to evaluate | Score | Description |
| --- | --- | --- |
| Head control | Supine, lifts head 45° | |
|  | 0 | Does not initiate neck flexion |
|  | 1 | Initiates neck flexion but does not lift head |
|  | 2 | Lift head <45° |
|  | 3 | Lifts head 45° |
|  | Prone, lifts head upright | |
|  | 0 | Does not initiate head lifting |
|  | 1 | Initiates head lifting, chin does not clear mat |
|  | 2 | Lifts head, does not attain upright, chin clears mat |
|  | 3 | Lifts head upright |
|  | Supine, hands gasped by examiner: pulls self to sitting with head control | |
|  | 0 | Does not initiate head control when pulled to sitting |
|  | 1 | Initiates head control when pulled to sitting |
|  | 2 | Assists with pulling to sitting, head control present part of the time |
|  | 3 | Pulls self to sitting with head control |
|  | Sit on mat, supported at thorax by therapist lifts head upright, maintains 3 seconds | |
|  | 0 | Does not initiate head lift |
|  | 1 | Initiates head lift |
|  | 2 | Lifts head, does not attain upright, holds 3 seconds |
|  | 3 | Lifts head upright, maintains 3 seconds |
| Rolling | Supine : Rolls to prone over right/left | |
|  | 0 | Does not initiate rolling |
|  | 1 | Initiates rolling |
|  | 2 | Rolls part way to prone |
|  | 3 | Rolls to prone over right/left side |
|  | Prone : Rolls to supine over right/left | |
|  | 0 | Does not initiate rolling |
|  | 1 | Initiates rolling |
|  | 2 | Rolls part way to supine |
|  | 3 | Rolls to supine over right/left side |

**Supplemenatary Table 2. Tracheostomy indications (N=33)**

| Indications | N(%) |
| --- | --- |
| Subglottic stenosis | 7 (21.2) |
| laryngomalacia | 8 (24.2) |
| Prolonged intubation due to bronchopulmonary dysplasia | 16 (48.5) |
| Congenital central hypoventilation syndrome | 2 (6.0) |

**Supplementary Table 3. Specific medical history on minor brain anomaly, GI surgery, and heart anomaly.**

|  |  | **Total**  **(N=165)** | **Tracheostomy**  **(N=33)** | | **No tracheostomy**  **(N=132)** |
| --- | --- | --- | --- | --- | --- |
| Minor brain abnormality | Total | 73 | | 19 | 54 |
|  | GM-IVH grade 1  GM-IVH grade 2 | 54  16 | | 12  7 | 42  9 |
| GI surgeries | Total | 70 | | 11 | 59 |
|  | Inguinal hernia repair  Intestinal resection  Esophageal atresia repair | 32  22  5 | | 3  4  1 | 29  18  4 |
| Heart anomaly† | Total | 80 | | 20 | 60 |
|  | Patent ductus arteriosus  Ventricular septal defect  Atrial septal defect  Pulmonary stenosis | 12  28  24  6 | | 5  7  6  2 | 7  21  18  4 |

Values are presented in number of subjects.
†Heart anomaly groups are composed only of those with spontaneous resolution, or those who went through management such as medication, or percutaneous catheter procedures. Those with open heart surgeries were not included in this study.

GI, Gastrointestinal; GM-IVH, Germinal matrix- intraventricular hemorrhage.
